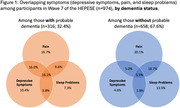# Sex/Gender Differences in the Pattern of Comorbid Depressive Symptoms, Pain, and Sleep Problems among Older Mexican Americans With and Without Probable Dementia

**DOI:** 10.1002/alz.090477

**Published:** 2025-01-09

**Authors:** Sadaf Arefi Milani, Trinity Brigham, Yong‐ Fang Kuo, Brian Downer, Kyriakos S. Markides, Mukaila A Raji

**Affiliations:** ^1^ University of Texas Medical Branch, Galveston, TX USA

## Abstract

**Background:**

Depression, pain, and sleep problems commonly co‐occur (overlap) among older adults. Data are lacking on the extent, pattern and sex/gender differences of overlap of these symptoms in adults aged ≥80 and living with dementia. Our objective was to examine patterns and sex/gender differences in overlapping depression‐pain‐sleep symptoms among older Mexican Americans with and without probable dementia.

**Method:**

We used data from Wave 7 (2010/2011) of the Hispanic Established Population for the Epidemiologic Study of the Elderly, a study of Mexican Americans aged ≥75, residing in the Southwestern US. Participants were considered to have probable dementia if they scored <21 on the Mini‐Mental State Examination and had ≥1 activity of daily living limitation. Pain was defined as pain on weight‐bearing. Depressive symptoms were defined as having scores of 16 or more on the Center for Epidemiologic Studies Depression Scale. Clinically relevant sleep problems were defined as having trouble falling asleep, waking up several times per night, trouble staying asleep, or waking up feeling tired for at least 15 days in the past month. We used descriptive statistics to describe the prevalence and overlap in these three symptoms, by dementia status and sex/gender.

**Result:**

Our sample was 85.7 years old on average and 65% were women (n = 974). Almost one‐third of participants (32.4%) had dementia. About 16% of participants with probable dementia reported overlapping depression‐pain‐sleep symptoms, compared to 5.5% of those without dementia (Figure 1). When stratified by cognitive status and sex/gender, 14.3% of men and 17.0% of women with probable dementia reported all three symptoms, compared to 2.0% of men and 7.6% of women without dementia.

**Conclusion:**

Participants with probable dementia reported three times the frequency of co‐occurrence of clinically relevant depressive symptoms, pain, and sleep problems compared to those without dementia. Women, regardless of cognitive status, more frequently reported depression‐pain‐sleep symptom overlap, compared to men, but the gender disparity was smaller for those with probable dementia. The presence of one symptom should alert clinicians to screen for, treat, and manage the other two. Evidence supports common underlying mechanisms for these three highly co‐occurring conditions, potentially informing therapeutic decision‐making in patients with dementia.